# Menthol cigarettes and smoking cessation behavior

**DOI:** 10.1186/1617-9625-9-S1-S6

**Published:** 2011-05-23

**Authors:** Allison C Hoffman, Donna Miceli

**Affiliations:** 1 Center for Tobacco Products, Food and Drug Administration, Rockville, MD 20850, USA; 2 Freelance medical write, Fort Myers, FL, USA

## Abstract

Although much is known about smoking cessation behavior, the vast majority of research has not assessed menthol as an independent factor. The objective of this review is to assess the effects, if any, that use of menthol cigarettes has on smoking cessation success in adults and youth. A total of 20 articles are included in this review. Although some studies have found that menthol smokers have less success in quitting smoking, others fail to find significant differences between menthol and non-menthol smokers. Some clinical trials evaluating the efficacy of various cessation treatments have suggested that menthol smokers have poorer outcomes, however two secondary data analysis studies (which used the same original dataset) failed to find any difference in success rate associated with particular treatments. Although there is some suggestion that smoking menthol cigarettes is associated with worse cessation outcomes, differences are not always found. However, if there was a difference, it was always in the direction of worse outcomes for menthol smokers. Given that Black/African American smokers prefer menthol cigarettes more than White smokers, possible interactions with race/ethnicity are discussed.

## Introduction

According to the Centers for Disease Control and Prevention, 70% of current U.S. adult smokers report that they want to quit completely, and the vast majority of smokers have attempted to quit smoking at least once [1]. Although much is known about tobacco cessation behavior in general, much less is known about the role that menthol may play in cessation behavior and success. Since a significant number of smokers in the United States smoke menthol cigarettes, including 68.9% of Black smokers, 29.2% of Hispanic smokers, and 22.4% of White smokers [2], and menthol smokers are less likely to be former smokers [3], this a significant public health question. This review summarizes the current literature regarding the possible relationship between smoking menthol cigarettes and cessation. Questions addressed include:

• What role, if any, does menthol play in smoking cessation and treatment outcomes in adults?

• What interactions, if any, does menthol have with ethnicity/race in smoking cessation success?

• What role, if any, does menthol play in smoking cessation and treatment outcomes in youth?

## Method

Summarized in this review are 20 articles found to have either direct relevance to these questions, or used to provide relevant background information. Many of these articles were identified through a review of the literature conducted by the National Cancer Institute in 2009, published as “Bibliography of literature on menthol and tobacco.” (http://cancercontrol.cancer.gov/tcrb/documents/menthol_bibliography_508.pdf). Search terms used were menthol cigarette(s); mentholated cigarette(s); menthol tobacco; mentholated tobacco; menthol smoker(s); menthol AND the following terms: addiction, nicotine, marketing, cancer, biomarkers, asthma, cardiovascular disease, heart disease, vascular disease, chronic obstructive lung disease, respiratory, environmental tobacco smoke, national health, health disparities, and minority health. Additional searches and sources, such as those identified through review articles, identified additional articles that were included as appropriate. A publication or study is identified as having a tobacco industry association if one or more authors were employees of the tobacco industry, as identified by author affiliation on the publication, however there are no such studies cited in this paper.

Of those articles that are in the NCI Bibliography but were not included, most were not directly relevant to this topic (e.g., they studied menthol as a chemical independent from tobacco smoke exposure, did not evaluate menthol as a separate variable). Some of those articles, however, were used to provide background information. Animal or in vitro research was included only to help explain human findings. Although a few review articles were used to make general statements and/or provide background information, most were not included in deference to original sources. Published abstracts were not included out of concern that, due to the lack of details, those studies could not adequately be assessed.

## Results

### Menthol and smoking cessation among adults

Five studies assessed smoking patterns in adult menthol smokers, particularly Black/African American smokers and whether smoking menthol cigarettes altered the likelihood of successful quitting. There were many studies that compared cessation success among Black/African American smokers and White smokers, but did not evaluate the independent effect of menthol cigarette use. Therefore, although menthol may have been postulated as a potential factor influencing success, these studies are not included in this review.

Hyland et al [4] analyzed the Community Intervention Trial for Smoking Cessation (COMMIT) dataset to assess whether use of menthol cigarettes was associated with quitting. This large-scale telephone survey was first completed in 1988, with a follow up (re-interview) in 1993. No association between smoking menthol cigarettes and quitting success was found.

Muscat et al [5] found similar results in a cross-sectional analysis of case-control data from a study conducted on more than 19,000 current and former cigarette smokers. The purpose of the study was to determine if the menthol cigarettes were associated with the frequency of smoking and with quitting, and whether cigarette mentholation explains racial differÂ¬ences in these two smoking behaviors. The authors concluded that the likelihood of cessation was not associated with the preference for menthol cigarettes.

In an effort to better understand the smoking patterns and cessation experiences of Black/African American smokers, Okuyemi et al [6] conducted a cross-sectional survey of 600 Black/African American smokers at an inner-city health center. To examine past cessation attempts, survey questions focused on the number of lifetime quit attempts, time since most recent quit attempt, and the duration of abstinence for both the longest-ever and most recent quit attempts. There was no difference between menthol and non-menthol smokers in the number of lifetime quit attempts, but menthol smokers had significantly less time since their last quit attempt. Additionally, there was a suggestion that smokers of menthol cigarettes had shorter durations of abstinence for both their most recent and their longest-ever quit attempts, but the results were not statistically significant (p = 0.187 and p = 0.111, respectively). Based on the consistency of the direction of the three measures of cessation success, the authors suggested that Black/African American individuals who smoke menthol cigarettes may be less likely to be successful in their quit attempts.

Fagan and colleagues [7] used multivariate models to assess possible differences in duration of smoking abstinence in 46,273 adult tobacco users. Secondary analysis of data from the cross-sectional 2003 and 2006/7 Tobacco Use Supplements to the Current Population Surveys (TUS-CPS) failed to find differences in duration of smoking abstinence >2 weeks during the past 12 months when comparing menthol to non-menthol smokers.

#### Menthol’s effect on smoking cessation among adults using cessation treatments

The seven articles discussed in this section deal specifically with menthol’s effect on quitting rates among persons using smoking cessation interventions (e.g., bupropion, nicotine gum, counseling). They focused primarily on Black/African American smokers. Studies that did not specifically investigate the role of menthol in quitting success were not included in this review.

In an attempt to better understand the association between menthol cigarette use and cessation in Black/African American smokers, Okuyemi et al [8] analyzed data from the first double-blind, placebo-controlled, randomized trial of bupropion in Black/African-American smokers [9] and reported that although bupropion increased successful abstinence, the 7-day point prevalence of abstinence for menthol smokers was only half that of non-menthol smokers (36.2% compared to 60.3%). Overall, at the 6-week follow-up, menthol smokers were only half as likely to remain abstinent as compared to non-menthol smokers (24.9% and 44.4%, respectively). These findings suggest that smoking menthol cigarettes contributes to difficulty in remaining abstinent while using bupropion as a cessation aid.

Results from a study designed to identify individual characteristics that predict successful smoking cessation treatment among Black/African American smokers demonstrated that, other than bupropion treatment, the strongest predictors for success included not smoking menthol cigarettes [10]. This study involved an analysis of 21 baseline variables from the randomized controlled trial of bupropion cited previously [9]. Results from a univariate analysis demonstrated that fewer Black/African American individuals who smoked menthol cigarettes successfully quit (28.3% vs. 41.54%). According to the authors, this study may have been limited by the fact that the participants were primarily female and middle aged. When paired with Okuyemi et al [8], these results support the hypotheses that Black/African American smokers of menthol cigarettes are less likely to be successful in their quit attempts than Black/African American smokers of non-menthol cigarettes.

A cohort analysis of 1,021 patients in a tobacco dependence clinic who were treated with combinations of counseling (individual and/or group) and a range of cessation medications also found that menthol smokers were less successful in quitting than non-menthol smokers. Compared to non-menthol smokers, patients who reported that their current cigarette brand was a menthol cigarette were significantly less likely to be abstinent at 4 weeks (35.4% compared to 52.3%; p < .00001) and at 26 weeks (24.9% compared to 35.8%; p < .00001). Length of treatment has been shown to be positively correlated with treatment outcomes, with longer treatment times associated with better treatment outcomes. In this study, treatment time was variable; however, analyses regarding possible differences in treatment time for menthol and non-menthol smokers were not reported. Thus, if menthol smokers participated in treatment for a shorter period of time, that may have reduced their relative likelihood of success [11].

Okuyemi et al [12] also reported on the relationship between smoking menthol cigarettes and smoking cessation among Black/African American smokers who smoked fewer than 10 cigarettes per day (CPD) (“light” smokers), using data from a clinical trial that assessed the efficacy of 2 mg nicotine gum versus placebo, and motivational interviewing (MI) versus health education (HE). The 615 light smokers of menthol cigarettes who were enrolled in the trial were evenly distributed across four study groups. The results from this double-blind, placebo-controlled study demonstrated that, similar to the findings of cessation studies involving moderate-to-heavy smokers [8-10], quitting rates at 26 weeks after enrollment were lower for Black/African American light smokers who smoked menthol cigarettes than for Black/African American light smokers of non-menthol cigarettes. In addition, the effect of menthol cigarette use on cessation was limited to individuals less than 50 years old, which is also consistent with the findings of the previous studies. The authors pointed out that these results were significant because the study not only involved light smokers, but also used different forms of intervention (gum and counseling). They further suggested that because the majority of Black/African American smokers use menthol cigarettes, there is a need for a better understanding of the mechanism behind this lower quit rate.

Female prisoners in a clinical trial studying smoking cessation treatment were given 10 weeks of treatment (group psychotherapy and nicotine replacement therapy) and followed for 12 months [13]. At the 12-month follow-up, there were no significant differences in abstinence rates when menthol smokers were compared to non-menthol smokers. There are a few caveats when interpreting these data, however. Preference for menthol versus non-menthol was assessed during incarceration only and may be different than cigarette preference pre-incarceration. Additionally, some of the groups were very small (e.g., Black/African American non-menthol smokers, n = 3) while others were comparatively large (e.g., Black/African American menthol smokers, n = 121), which may have skewed the results.

#### Multi-ethnic differences in smoking cessation among menthol smokers

The seven articles reviewed in this section focus on racial and ethnic differences in smoking cessation and the impact menthol may have on quit attempts.

The Coronary Artery Risk Development in Young Adults (CARDIA) study [14] is a longitudinal study that prospectively measured cumulative exposure to menthol and non-menthol cigarettes and smoking cessation behavior from 1985 to 2000 among 5,115 Black/African American and European American smokers. Although the findings supported the premise that individuals who smoke menthol cigarettes are less likely to try to quit and more likely to relapse than those who smoke non-menthol cigarettes, these results were not statistically significant.

In two studies, Fu et al [15,16] examined the effect of menthol cigarette smoking on cessation among a multi-ethnic sample of smokers who attempted to quit smoking. These studies involved secondary analysis of data from a multisite randomized, controlled trial, and included more than 1,300 participants aged 19 years or older from the Department of Veterans Affairs pharmacy database. Twenty-five percent (343 out of 1,343) of this multi-ethnic sample smoked menthol cigarettes. Results demonstrated no significant association between smoking menthol cigarettes and a lower rate of subsequent cessation from cigarettes. In addition, there were no significant ethnic differences in smoking cessation rates.

Similar results were found in a clinical trial of smoking cessation among female prisoners. Using data from a smoking cessation intervention, Cropsey et al [13] compared White and Black/African-American female prisoners who were participating in a 10-week intervention involving group psychotherapy and the nicotine replacement patch. Although both groups of smokers benefited from the cessation treatment compared with the untreated control group, over time, the success rate was significantly higher among White smokers compared with Black/African American smokers. Although Black/African American smokers were more than twice as likely as White smokers to smoke menthol cigarettes while incarcerated (80.2% vs. 38.7%), menthol preference was not associated with differences in quit rates. It is important to note that some cell sizes, especially those of Black/African-American non-menthol smokers, are small, and thus caution should be used when interpreting the data. Also, some smokers changed their preference for menthol versus non-menthol cigarettes while in prison, which may have implications for the results.

Gandhi et al [17] conducted a retrospective cohort analysis of 1,688 consecutive patients who attempted to quit smoking using multidisciplinary treatment options. At the 4-week follow-up visit, quit rates among White, Black/African American, and Hispanic/Latino smokers of menthol cigarettes were lower than the quit rates of those in the same racial/ethnic subgroups who smoked non-menthol cigarettes. At the 6-month follow-up visit, quit rates among Black/African American menthol cigarette smokers remained significantly lower than the quit rates of Black/African American smokers of non-menthol cigarettes, even after controlling for factors associated with cessation. Although there were similar patterns seen in Hispanic smokers, they did not reach statistical significance. At 6 months, there were no differences in cessation success between White menthol and non-menthol smokers.

In a study designed to explore the relationship between race, ethnicity, menthol smoking, and cessation, Gundersen et al [3] found some support for the hypothesis that smoking menthol cigarettes can result in poorer cessation outcomes, but these results were only found for the non-White population. The authors analyzed data from 7,815 White, Black/African American, and Hispanic/Latino current smokers in the 2005 U.S. National Health Interview Survey who indicated they did not currently use other tobacco products and had made quit attempts. Those who had smoked at least 100 cigarettes but were no longer smoking at the time of data collection were considered former smokers. After controlling for demographic factors and smoking behavior (e.g., CPD), non-White (Black/African American and Hispanic/Latino) menthol smokers were significantly less likely to quit than non-White non-menthol smokers. Interestingly, White menthol smokers were more likely to be former smokers as compared to White non-menthol smokers. In their conclusion, the authors stressed the need for further research designed to understand the mechanisms by which smoking menthol cigarettes impacts cessation and why it may have differing effects among White smokers and non-White smokers.

Using the 2003-2006/7 TUS-CPS dataset, Trinidad and colleagues [18] examined cross-sectional data from 54,818 former smokers, between 20 and 65 years of age. Using adjusted logistic regression models predicting successful smoking cessation of greater than 6 months, African Americans (OR = 0.23, 95% CI: 0.17–0.31), Asian Americans/Pacific Islanders (OR = 0.22, 95% CI: 0.11–0.45), Hispanics/Latinos (OR = 0.48, 95% CI: 0.34–0.69), and non-Hispanic whites (OR = 0.28, 95% CI:0.25–0.33) who smoked menthol cigarettes were significantly less likely to have quit successfully. Among Native Americans/Alaska Native former smokers, there were no significant differences between menthol and non-menthol former smokers (OR = 0.49, 95% CI: 0.14–1.71).

Using data from the cross-sectional 2005 National Health Interview Survey (NHIS), Stahre and colleagues [19] examined data from current (n=6511) and former (n=6774) smokers. After controlling for other factors, multiple regression analysis found a significant difference in the quit ratio for menthol versus non-menthol among African American smokers (34% versus 49%, P < 0.001). The authors conclude that race significantly modifies the effect of menthol cigarette smoking on smoking cessation as measured by the population quit ratio, with African American menthol cigarette smoking being associated with decreased likelihood of smoking cessation.

### Menthol and smoking cessation among youth

Although many of the studies discussed in this paper alluded to the popularity of menthol cigarettes among young people, there were no research studies that specifically focused on menthol cigarette use and youth cessation outcomes; however, data from the 2002 National Youth Tobacco Survey suggests differences in intent to quit, with adolescent menthol smokers being significantly less likely to report “seriously thinking about quitting” (p < .05) [20]. Those who tried to quit, however, were more likely to seek help in quitting, such as using nicotine gum, attending a school program, visiting an Internet cessation site or calling a cessation helpline [20].

## Summary/discussion

There were several studies that found no association between adult use of menthol cigarettes and cessation success, including a national survey [4], a local/regional survey [6], a longitudinal study [14] a clinical study [13], secondary data analysis of a large-scale randomized intervention study [15], and secondary analysis of a large cross-sectional survey [7]. There were also several studies that found that adult menthol smokers have lower levels of abstinence/successful quitting, including several clinical studies with both moderate/heavy smokers and light smoker, [6,10] and in a national survey [3]. There were some clinical and cohort analysis studies that found that efficacious behavioral and pharmacotherapy treatments were less effective when used by menthol smokers as compared to non-menthol smokers [7,11,12], however two secondary data analysis studies (which shared the same original data) failed to find differences in cessation success [15,16]. In addition, a possible interaction between menthol and race/ethnicity was suggested, with worse outcomes for adult Black/African American and/or Hispanic/Latino menthol smokers than White menthol smokers [3,16,18,19]. There was no consistent interaction between menthol cigarette use and quitting success for White smokers.

## Competing interests

The authors declare that they have no competing interests.

## Supplementary Material

Additional file 1 “Cessation Reference Table” This file is a table summarizing the included studies with the author(s), title, publication year, type of study, subject description and selected author conclusions. Click here for file
