# Supplementary material for: Menthol cigarettes and smoking cessation behavior
Source: Tob Induc Dis. 2011 May 23;9(Suppl 1):S6. doi: 10.1186/1617-9625-9-S1-S6 (PMC3102905; doi:10.1186/1617-9625-9-S1-S6)
Supplement: Additional file 1 — “Cessation Reference Table” This file is a table summarizing the included studies with the author(s), title, publication year, type of study, subject description and selected author conclusions. [file 1617-9625-9-S1-S6-S1.pdf]

Cessation Reference Table

Author Name(s)	Article Title	Pub. Year	Type of Study	Subject Description	Selected Author's Conclusions
Ahluwalia JS, Harris KJ, Catley D, et al	Sustained-release bupropion smoking cessation in African Americans—a randomized controlled trial	2002	Randomized, Double Blind, Placebo controlled	African American adults who smoked 10 or more cigarettes per day. There were 600 participants (placebo = 300; buproprion treatment = 300)	There is some evidence that smokers using menthol cigarettes may be less likely to quit than those not smoking these types of cigarettes.
Centers for Disease Control and Prevention	Cigarette smoking among adults—United States, 2000	2002	Survey (questionnaire, personal interview)	Adults ≥18 years who were current smokers (US, non-institutionalized, civilian). N=32,374 surveyed; the survey response rate was 72.1%.	The findings in this report indicate that current smoking prevalence has remained stable among persons aged 18–24 years.
Cropsey KL, Weaver MF, Eldridge GD, et al.	Differential success rates in racial groups: results of a clinical trial of smoking cessation among female prisoners	2009	Randomized case control trial.	White and Black female prisoners, aged ≥18, smoking at least 5 cpd. N=233 cases; N= 289 controls	Smoking menthol cigarettes was not associated with differences in quit rates.
Fagan, P, Moolchan, ET, Hart A, et al.	Nicotine dependence and quitting behaviors among menthol and non-menthol smokers with similar consumptive patterns	2010	The 2003 and 2006/07 Tobacco Use Supplements to the Current Population Surveys	Daily current smokers aged 18+ (N = 46,273).	There were no associations between menthol versus non-menthol smokers in duration of smoking abstinence.

Foulds JF, Gandhi KK, Steinberg MB, et al	Factors associated with quitting smoking at a tobacco treatment dependence treatment clinic	2006	Cohort Study	1021 patients who attempted to quit tobacco at a specialist tobacco dependence treatment outpatient clinic based at the Tobacco Dependence Program at the University of Medicine and Dentistry of New Jersey-School of Public Health	Forty-one percent of the patients smoked menthol cigarettes. They were less likely to achieve abstinence in univariate analyses, and this item remained in the model predicting 4-week outcome.
Fu SS, Kodl MM, Joseph AM, et al	Racial/ethnic disparities in the use of nicotine replacement therapy and quit ratios in lifetime smokers ages 25 to 44 years	2008	Large, randomized intervention study	Adults ages 25 - 44 years from 3 metropolitan areas in the Midwest were randomly sampled using Health Maintenance Organization membership lists in Detroit, MI and Minneapolis, MN and a driver's license registry in St. Louis, MO; sample was limited to lifetime smokers (individuals who had ever smoked >100 cigarettes). There were 27,031 participants, including Caucasian (n= 7,907), African American (n= 955), Latino (n= 246), and Asian (n=108) smokers.	No association between adult use of menthol cigarettes and cessation success.
Fu, SS, Okuyemi, KS, Partin, MR et al.	Menthol cigarettes and smoking cessation during an aided quit attempt	2008	Secondary analysis of randomized, controlled intervention trial	Participants were identified from the Department of Veterans Affairs (VA) pharmacy databases and included smokers with a recent quit attempt that incorporated pharmacologic treatment (N = 51,343)	There was no significant effect of smoking menthol cigarettes on aided quit attempts.

Gandhi KK, Foulds J, Steinberg MM, et al	Lower quit rates among African American and Latino menthol cigarette smokers at a tobacco treatment clinic	2009	Retrospective Cohort/ Population Studies	Specialized smoking cessation outpatient clinic in New Jersey: patients who set a quit date and attempted to quit smoking, between 1/1/2001 and 6/30/2005; A total of 1688 African American, Latino and Whites participants.	This study found lower short-term (4-week follow-up) quit rates among African American and Latino menthol smokers as compared with non-menthol smokers within the same racial⁄ethnic subgroups.
Giovino GA, Sidney S, Gfroerer JC, et al.	Epidemiology of menthol cigarette use	2004	Evaluation Survey	Not applicable	Available data indicate that mentholated cigarettes are at least as dangerous as their non-mentholated counterparts. The authors suggest that because mentholation improves the taste of cigarettes for a substantial segment of the smoking population and appears to mask disease symptoms, this additive may facilitate initiation or inhibit quitting.
Gundersen DA, Delnevo CD, Wackowski O	Exploring the relationship between race/ethnicity, menthol smoking, and cessation, in a nationally representative sample of adults	2009	Retrospective analysis of cross-sectional study	Sample of those who indicated that they do not currently use other tobacco products and have made a quit attempt (N=7815).	Menthol smoking can lead to poorer cessation outcomes, but only for non-white smokers.
Harris KJ, Okuyemi KS, Catley D, et al	Predictors of smoking cessation among African-Americans enrolled in a randomized controlled trial of bupropion.	2004	Double-blind placebo-controlled, randomized trial	Of the 1,498 smokers in a mid-western city who identified themselves as black or African-American that were screened, 981 were eligible and invited to participate, and 600 participated.	Other than bupropion treatment, the strongest predictors for success included not smoking menthol cigarettes.

Hersey JC, Ng SW, et al	Are menthol cigarettes a starter product for youth?	2006	2000 and 2002 NYTS, School-based, national survey	The survey used a three-stage cluster sample design that oversampled African American, Hispanic, and Asian students. The NYTS was administered to35,828 students in grades 6 through 12 in spring 2000 and to 26,149 students in spring 2002. N=5,512 youth (2000 NYTS) and 3,202 youth (2002 NYTS)	Adolescent menthol smokers were significantly less likely to report "seriously thinking about quitting" ($p < .05$). Menthol smokers were more likely to seek help in quitting, such as using nicotine gum, attending a school program, visiting an Internet cessation site or calling a cessation helpline.
Hyland A, Garten S, Cummings KM	Mentholated cigarettes and smoking cessation: findings from COMMIT (Community Intervention Trial for Smoking Cessation)	2002	Telephone survey	COMMIT study: Baseline smokers who reported whether their current cigarette brand or not in 1988, and had a known smoking status in 1993. N=13,268 (3,184 menthol, 10084 non-menthol)	No clear associations were observed between menthol cigarette use and indicators of nicotine dependence, even after controlling for race/ethnicity and other demographics.
Muscat JE, Richie JP Jr, et al.	Mentholated cigarettes and smoking habits in Whites and Blacks	2002	Cohort/ Population	Hospital between 1981 and 1999: 19 545 subjects. Eleven per cent of subjects were black, including 16,540 (84.6%) smokers of non-mentholated cigarettes and 3005 (15.4%) smokers of mentholated cigarettes.	There were no differences in quitting associated with use of menthol cigarettes.
Okuyemi KS, Ahluwalia JS, Ebersole-Robinson M, et al	Does menthol attenuate the effect of bupropion among African American smokers?	2003	Randomized Controlled Trial	African American smokers enrolled in a clinical trial that assessed the efficacy of sustained-release bupropion for smoking cessation. A total of 600 participants (471 menthol smokers and 129 non-menthol smokers).	African American menthol smokers had lower smoking cessation rates after 6 weeks of treatment with bupropion-SR than African-American non-menthol smokers. According to the authors, this suggests that menthol smokers are at greater risk from the health effects of smoking.

Okuyemi KS, Ebersole-Robinson M, Nazir N, et al.	African-American menthol and non-menthol smokers: differences in smoking and cessation experiences	2004	Cross sectional survey	African-American smokers at an inner-city health center. Menthol smokers (n = 407) were compared to non-menthol smokers (n = 73).	There were no significant differences in the individual measures of cessation success. However, based on the consistency of the direction of the three measures of cessation success, the authors suggested that Black/African American individuals who smoke menthol cigarettes may be less likely to be successful in their quit attempts.
Okuyemi KS, Faseru B, Cox LS, et al.	Relationship between menthol cigarettes and smoking cessation among African American light smokers	2007	Randomized Controlled Trial	755 African American light smokers (≤ 10 cpd)	Among African American light smokers, use of menthol cigarettes is associated with lower smoking cessation rates.
Pletcher MJ, Hulley BJ, Houston T, et al.	Menthol cigarettes, smoking cessation, atherosclerosis, and pulmonary function: the Coronary Artery Risk Development in Young Adults (CARDIA) study	2006	Multi-center U.S. cohort study	African American and European American smokers aged 18 to 30 years and healthy at the time of enrollment, including 1544 participants (563 non-menthol smokers; 972 menthol smokers)	There was a trend towards menthol smokers having lower cessation rates, but this failed to reach significance.
Stahre, M, Okuyemi, KS, Joseph, AM, et al	Racial/ethnic differences in menthol cigarette smoking, population quit ratios and utilization of evidence-based tobacco cessation treatments	2010	Secondary data analysis of cross-sectional data from the 2005 National Health Interview Survey (NHIS) Cancer Control Supplement.	The NHIS is a cross-sectional survey of nationally representative non-institutionalized adults. The 2005 Cancer Control Supplement collected information related to a variety of cancer-related health indicators, including smoking and cessation utilization on a subsample of the adult population. Participants included 31,428 people aged 18 and older. Approximately 42% of adults	Compared to white non-menthol smokers, African American menthol smokers were significantly less likely than have quit smoking (adjusted odds ratio: 0.72, 95% confidence interval: 0.53, 0.97) after controlling for numerous variables. Authors conclude that menthol cigarette smoking is associated negatively with successful smoking cessation among African Americans.

				reported being either a current (*n* = 6511) or former (n = 6774) smoker.	
Trinidad, DR, Perez-Stable, EJ, Messer K, et al	Menthol cigarettes and smoking cessation among racial/ethnic groups in the United States	2010	Secondary data analysis of the 2003 and 2006–07 Tobacco Use Supplements to the Current Population Survey (TUS CPS)	Six waves of the tobacco use supplements of the TUS CPS were included. Participants included 125,369 people between 20 and 65 years of age at the time of survey (ever smokers, current smokers, and former smokers).	Among former smokers, those who used to smoke mentholated cigarettes regularly were less likely to have experienced long-term quitting success. African Americans and Hispanics/Latinos who smoked mentholated cigarettes were less likely to quit successfully for at least 6 months compared to those who smoked non-mentholated cigarettes.
